# Supplementary material for: ProkBERT family: genomic language models for microbiome applications
Source: Front Microbiol. 2024 Jan 12;14:1331233. doi: 10.3389/fmicb.2023.1331233 (PMC10810988; doi:10.3389/fmicb.2023.1331233)
Supplement: Supplementary file 1 [file Data_Sheet_1.PDF]

ProkBERT Family: Genomic Language Models for Microbiome Applications

1 SUPPLEMENTARY INFORMATION

1.1 ProkBERT and transformer model architecture

The Bidirectional Encoder Representations from Transformers (BERT) architecture fundamentally relies on the transformer encoder mechanism. Unlike traditional models that process input data in a sequential manner, BERT's encoder reads the entire sequence of tokens at once, thus enabling bidirectional context understanding Devlin et al. (2019).

Each input token is first converted into a vector through an embedding layer. The core of the BERT encoder consists of multiple identical layers, each containing two sub-layers: a multi-head self-attention mechanism, and a simple, position-wise fully connected feed-forward network Vaswani et al. (2017). This design allows each token to be influenced by others in the sequence, facilitating a deeper contextual representation. Mathematically, the input for each token in a sequence is represented as:

$$\mathbf{h}_i^{(l)} = \text{TransformerEncoder}(\mathbf{h}_i^{(l-1)}), \quad i = 1, \ldots, n \tag{S1}$$

where $\mathbf{h}_i^{(l)}$ is the hidden state of token i at layer l, and n is the number of tokens in the input sequence. The output of the final layer $\mathbf{h}_i^{(L)}$, where L is the total number of layers, is used as the representation of each token. This representation encapsulates both left and right context of each token in the sequence, leading to a more nuanced understanding of the text.

In our paper, we presented three potential applications of ProkBERT, although the versatility of the encoders allows for numerous other possibilities. Figure S1 summarizes the model architecture and its possible application usages applied in this paper. nitially, the input sequence data is tokenized into k-mers using LCA tokenization. Next, the ProkBERT encoder generates a representative vector for each token, as previously described. These vectors are typically processed by a 'head' which may be designed for classification, masking, or pooling tasks. This head is usually simple and has a low complexity. For pretraining, a masked head was utilized, as detailed in Section 2.2 of the main text. For the results presented in Section 3.1 of the main paper we applied a pooling, as described in Section 2.2.4. Finally, fine-tuning was performed using a straightforward classification head, as outlined in Section 2.3.2 of the main text. Note, however, that there are many possible further applications. For example, it is straightforward to define token classification tasks where the model assigns a label to each token. Similarly, regression tasks, such as predicting gene expression, can be implemented. Additionally, it is also possible to combine the model's output with other, non-deep learning approaches, such as XGBoost (Chen and Guestrin, 2016).

2 SUPPLEMENTARY TABLES AND FIGURES

2.1 Figures

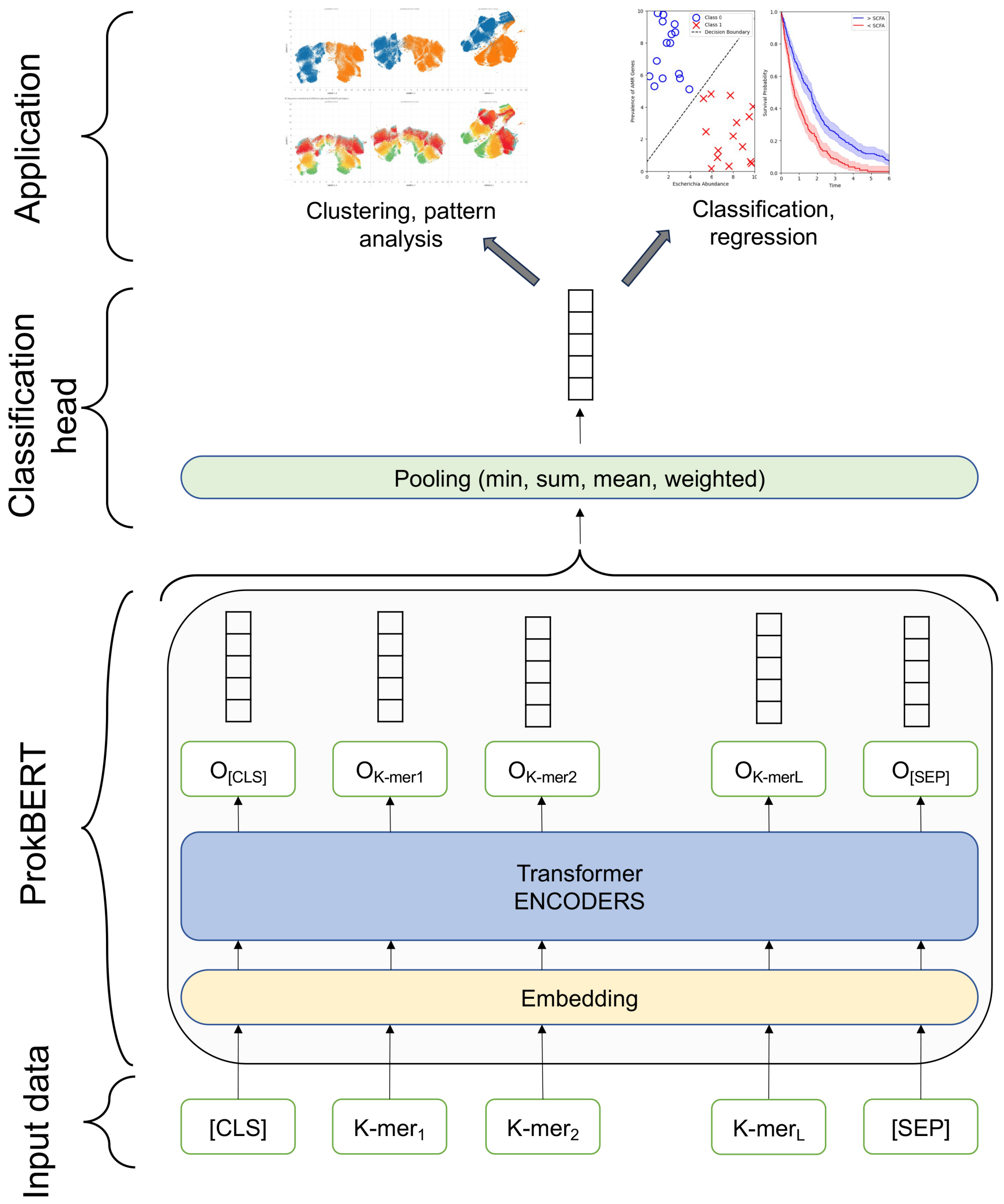

Figure S1. ProkBERT use cases. ProkBERT application overview. The prokBERT can be flexible use in various applications, for both supervised (i.e. binary classification or regression) and unsupervised learning cases (i.e. clustering the pattern analysis). First the input is encoded by the LCA tokenizer,input data tokens [CLS], K-mer$_1$, K-mer$_2$, ..., K-mer$_L$, [SEP] are first embedded into vectors and then processed through a series of Transformer encoder layers. The output vectors O_{CLS}, $O_{\text{K-mer}_1}$, $O_{\text{K-mer}_2}$, ..., $O_{\text{K-mer}_L}$, O_{SEP} from the top transformer layer are subjected to i) pooling operations (min, sum, mean, weighted) to condense the information into a fixed-size vector, ii) a classification head (i.e. a regularized linear layer), iii) or a masking head decoding token probabilities from the vector representations.

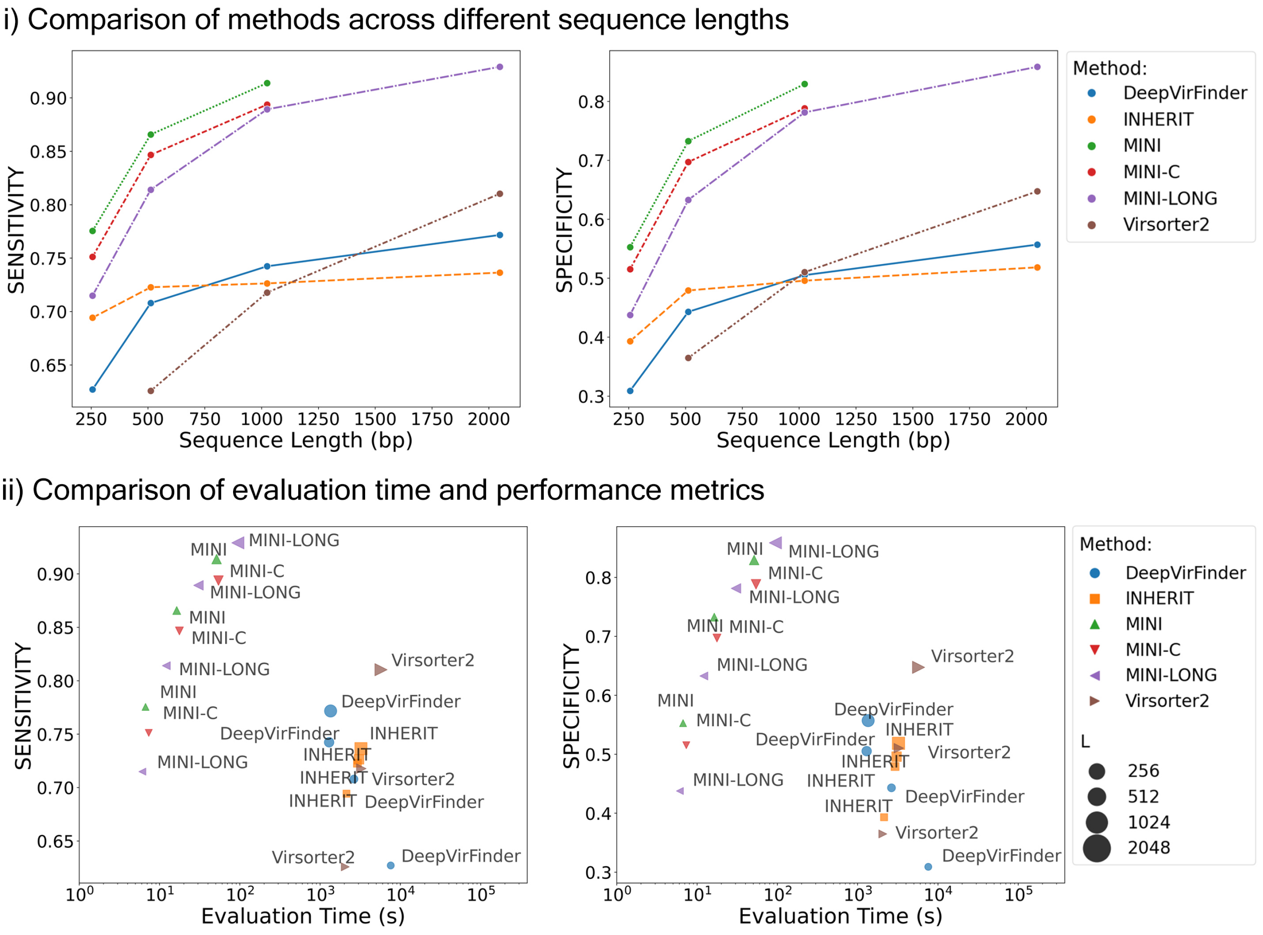

Figure S2. ProkBERT identifies phage sequences accurately and rapidly. i) Method comparison over varying sequence lengths based on two performance metrics: sensitivity, and specificity. ii) Scatter plots illustrating the relationship between evaluation time (on a logarithmic scale) and the mentioned performance metrics. The size of each point signifies the sequence length. Evaluation time encompasses model loading, sequence preprocessing, and inference phases.

REFERENCES

Chen, T. and Guestrin, C. (2016). XGBoost: A Scalable Tree Boosting System. In *Proceedings of the 22nd ACM SIGKDD International Conference on Knowledge Discovery and Data Mining*. 785–794. doi:10.1145/2939672.2939785

Devlin, J., Chang, M.-W., Lee, K., and Toutanova, K. (2019). BERT: Pre-training of Deep Bidirectional Transformers for Language Understanding. In *Proceedings of the 2019 Conference of the North American Chapter of the Association for Computational Linguistics: Human Language Technologies, Volume 1 (Long and Short Papers)*. 4171–4186

Vaswani, A., Shazeer, N., Parmar, N., Uszkoreit, J., Jones, L., Gomez, A. N., et al. (2017). Attention is all you need. *Advances in neural information processing systems* 30
